# Peripheral Inflammatory Hyperalgesia Depends on P2X7 Receptors in Satellite Glial Cells

**DOI:** 10.3389/fphys.2020.00473

**Published:** 2020-05-25

**Authors:** Amanda Ferreira Neves, Felipe Hertzing Farias, Silviane Fernandes de Magalhães, Dionéia Araldi, Marco Pagliusi, Claudia Herrera Tambeli, Cesar Renato Sartori, Celina Monteiro da Cruz Lotufo, Carlos Amílcar Parada

**Affiliations:** ^1^Department of Structural and Functional Biology, Institute of Biology, University of Campinas, Campinas, Brazil; ^2^Institute of Biomedical Sciences, Area of Physiological Sciences, Federal University of Uberlândia, Uberlândia, Brazil

**Keywords:** satellite glial cells, P2X7 receptors, interleukin-1beta, dorsal root ganglion, inflammatory hyperalgesia

## Abstract

Peripheral inflammatory hyperalgesia depends on the sensitization of primary nociceptive neurons. Inflammation drives molecular alterations not only locally but also in the dorsal root ganglion (DRG) where interleukin-1 beta (IL-1β) and purinoceptors are upregulated. Activation of the P2X7 purinoceptors by ATP is essential for IL-1β maturation and release. At the DRG, P2X7R are expressed by satellite glial cells (SGCs) surrounding sensory neurons soma. Although SGCs have no projections outside the sensory ganglia these cells affect pain signaling through intercellular communication. Therefore, here we investigated whether activation of P2X7R by ATP and the subsequent release of IL-1β in DRG participate in peripheral inflammatory hyperalgesia. Immunofluorescent images confirmed the expression of P2X7R and IL-1β in SGCs of the DRG. The function of P2X7R was then verified using a selective antagonist, A-740003, or antisense for P2X7R administered in the L5-DRG. Inflammation was induced by CFA, carrageenan, IL-1β, or PGE_2_ administered in rat’s hind paw. Blockage of P2X7R at the DRG reduced the mechanical hyperalgesia induced by CFA, and prevented the mechanical hyperalgesia induced by carrageenan or IL-1β, but not PGE_2_. It was also found an increase in P2X7 mRNA expression at the DRG after peripheral inflammation. IL-1β production was also increased by inflammatory stimuli *in vivo* and *in vitro*, using SGC-enriched cultures stimulated with LPS. In LPS-stimulated cultures, activation of P2X7R by BzATP induced the release of IL-1β, which was blocked by A-740003. In summary, our data suggest that peripheral inflammation leads to the activation of P2X7R expressed by SGCs at the DRG. Then, ATP-induced activation of P2X7R mediates the release of IL-1β from SGC. This evidence places the SGC as an active player in the establishment of peripheral inflammatory hyperalgesia and highlights the importance of the events in DRG for the treatment of inflammatory diseases.

## Introduction

In the nociceptive system, primary afferent neurons transduce the injury information at its peripheral endings and transmit the resulting signal to the central nervous system (CNS). At this path, the electric signal will pass through an important structure that is often ignored, the sensory ganglia, which can be a dorsal root ganglion (DRG) or trigeminal ganglia. At the sensory ganglia resides the soma of nociceptors and other somesthetic neurons as well as specialized glial cells, the satellite glial cells (SGC). Besides the important role in metabolic support, events at DRG are known to participate in the development of pathological pain (Devor, 1999; Esposito et al., 2019). Primary afferent neurons at the DRG do not interact with one another through synaptic contact. A layer of satellite glial cells (SGCs) tightly wrap each neuronal soma, which in turn is enclosed by connective tissue to create a physically isolated unit (Devor, 1999; Hanani, 2005; Pannese, 2010; Esposito et al., 2019). This singular structure suggests active participation of SGCs in the processing of sensory information by communicating with neurons (Hanani, 2005; Costa and Moreira Neto, 2015; Fan et al., 2019). Several studies have described intercellular communication in the sensory ganglia involving gap junctions (Huang et al., 2010), calcium-dependent somatic exocytosis of transmitters (Huang and Neher, 1996), and purinergic receptors also known as purinoceptors (Gu et al., 2010; Verderio and Matteoli, 2011). Purinergic signaling through ATP release at the DRG is suggested to mediate the development of chronic pain (for review see Kobayashi et al., 2013; Magni et al., 2018).

ATP is a ubiquitous molecule found in all tissues and cells and is released into the extracellular milieu in both physiological and pathological conditions (Fitz, 2007). Receptors activated by extracellular ATP include two families of the P2 purinoceptors: the ligand-gated ionotropic channels P2X and the metabotropic G protein-coupled P2Y receptors (Burnstock, 2016). Purinoceptors have a widespread tissue distribution, including peripheral (PNS) and CNS, and they are known to partake in pain mechanisms and inflammation (North, 2016; Magni et al., 2018). For example, activation of neuronal P2X3 receptors mediates acute nociception, and the microglial P2X4 and P2X7 receptors are associated with neuropathic and inflammatory pain (Toulme et al., 2010; Inoue and Tsuda, 2012; Fabbretti, 2013; Burnstock, 2016). Increasing evidence suggest ATP as a major transmitter released in the sensory ganglia (Zhang et al., 2007; Kobayashi et al., 2013; Goto et al., 2017). Therefore, the purinoceptors in DRG may have an important role boosting intercellular communication (Gu et al., 2010; Verderio and Matteoli, 2011; Huang et al., 2013), particularly for pain signaling (Jarvis, 2010; Hanani, 2012; Kobayashi et al., 2013; Lemes et al., 2018).

After peripheral tissue injury or inflammation, molecular alterations in the DRG are involved in the development of hyperexcitability of nociceptive neurons (Krames, 2014; Berta et al., 2017). Examples of those alterations include the upregulation of P2X receptors (Kushnir et al., 2011) and upregulation of the proinflammatory cytokine interleukin-1β (IL-1β) (Guo et al., 2007; Araldi et al., 2013). IL-1β was the first proinflammatory cytokine described to be involved in inflammatory pain and hyperalgesia (Ferreira et al., 1988). Several reports emphasize that a key process for IL-1β maturation and secretion is the ATP-induced activation of the purinergic P2X7 receptors (P2X7R), selectively expressed in macrophages and microglia (Ferrari et al., 2006; Clark et al., 2010; Stoffels et al., 2015; Giuliani et al., 2017). Within the P2X family, P2X7R has the highest threshold for ATP-induced activation and will trigger downstream mechanisms only when extracellular ATP reaches pathological concentration (North, 2002). Undoubtfully P2X7R stands out among other purinoceptors for playing a central role in several pathologic conditions (De Marchi et al., 2016), including inflammatory diseases (Savio et al., 2018) and neuropathic pain (Zhang et al., 2020). Besides, studies have reported that P2X7R are expressed in SGC within DRG (Kobayashi et al., 2005; Zhang et al., 2005), suggesting a role for visceral hyperalgesia (Liu et al., 2015) and acute nociception (Lemes et al., 2018). Therefore, in this study, we hypothesized that ATP-induced activation of P2X7R and the subsequent release of IL-1β by SGCs could be a major process in the DRG for the establishment of inflammatory hyperalgesia in the peripheral tissue.

## Materials and Methods

### Animals

Experiments were performed using 180–250 g Wistar rats obtained from the University of Campinas Multidisciplinary Center for Biological Investigation (UNICAMP-CEMIB). Animals were housed in plastic cages with soft bedding (four/cage) on a 12-h light/dark cycle (lights on at 06:00 A.M.), with controlled humidity (60–80%) and temperature (22–25°C). Food and water were available *ad libitum*. Experimental protocols were approved by the Committee on Animal Research of the University of Campinas (CEUA – UNICAMP, protocol number: 3022-1). Animal care and handling procedures were in accordance with International Association for the Study of Pain (IASP) guidelines for the use of animals in pain research (Zimmermann, 1983). A total of 90 rats (males) were used for *in vivo* experiments and molecular analysis, and other 10 animals (males and females) were used for the *in vitro* experiments. Based on previous studies from our group, in inflammatory models, pain sensitivity and cytokine expression change according to estrous cycle in females (Joseph et al., 2003; Torres-Chávez et al., 2011). However, sexual dimorphism is abolished upon removal of the hormonal factors. For this reason, we used cultures of DRG cells from both male and female rats. During the experiments, animals were simply randomized into treatments. All efforts were made to minimize animal discomfort and to reduce the number of animals used.

### Hyperalgesia Induction

Complete Freund’s adjuvant (CFA 50 μL/paw, #F5881, Sigma Aldrich, St. Louis, MO, United States), λ-carrageenan (100 μg/paw, #22049, Sigma Aldrich, St. Louis, MO, United States), Interleukin 1 beta (IL-1β, 0.5 pg/paw, National Institute of Biological Standards and Control, South Mimms, Hertfordshire, United Kingdom) or PGE_2_ (100 ng/paw, #P5640, Sigma Aldrich, St. Louis, MO, United States) were administered subcutaneously (intraplantar) in the rat’s hind paw (right side) which is within the peripheral field of the L5 DRG (Araldi et al., 2013). The mechanical stimulus was then applied to the same area to measure hyperalgesia by electronic von Frey test.

### *In vivo* Treatments

A potent selective antagonist for P2X7R (A-740003; Tocris Bioscience, Bristol, United Kingdom) was administrated in the L5 DRG (right side) immediately before intraplantar injection of the inflammatory agent (right hind paw). A-740003 was diluted in a vehicle solution of 10% dimethyl sulfoxide (DMSO) + 10% propylene glycol + 80% sterile saline (NaCl 0.9%) and administrated at doses of 0.01, 0.10, and 1.00 mM. The concentrations were calculated based on the effective antihyperalgesic dose of 142 mg/kg used for systemic administration (i.p.) in similar inflammatory pain-like behaviors models by Honore et al. (2006). For intraganglionar administration, using rats with approximately 0.2 kg, we calculated concentrations 10-, 100-, and 1000-times lower (0.028, 0.28, and 2.8 mg/6 μl), which corresponds to the doses of 0.01, 0.10, and 1.00 mM.

The antisense (AS) oligonucleotide (ODN) for P2X7R (TTTCCTTATAGTACTTGGC) or a mismatch sequence (MM, TTCCGTTAAAGAAGTAGGC) were diluted in sterile saline and administrated in the L5 DRG (right side, 30 μg/5 μl) once a day for 4 days to allow the knockdown of the P2X7R prior to the intraplantar injection of the inflammatory agent in the right hind paw. To demonstrate the relative expression of P2X7R was not altered solely by the repeated intraganglionar injections, we also used non-treated DRG (on the contralateral side of the inflammation) in the RT-qPCR analysis as a control for basal gene expression. All ganglionar treatments in this work were administered ipsilateral to the inflammation.

### Ganglionar Drug Administration

The intraganglionar injection technique was performed as previously described (Ferrari et al., 2007; Araldi et al., 2013). Briefly, rats were anesthetized by inhalation of 2–3% isoflurane and an ultra-fine needle (32 G) was inserted through a punctured skin toward the intervertebral space between L5 and L6 vertebrae. Smooth movements of the needle were performed until a paw flinch reflex was observed and 5 μL of solution was injected. The paw-flinch reflex was used as a sign that the needle tip has reached the distal nerve insertion of the L5 DRG. This ganglionar administration is restricted to the injected L5 DRG and it does not reach the opposite ganglion, nor the spinal cord between L1-T13 segments (Oliveira et al., 2009).

### Mechanical Hyperalgesia Evaluation by Electronic von Frey Test

The withdrawal threshold of the treated hind paw was measured using an electronic von Frey aesthesiometer (Insight, Ribeirão Preto, SP, Brazil) as previously described (Vivancos et al., 2004). All experiments were performed by the same experimenter blind to all treatments, between 9:00 AM and 4:00 PM. Rats were kept in a quiet room for 1 h prior to any manipulation. Then, each animal was placed in an acrylic cage (12 cm × 20 cm × 17 cm) with a wire grid floor providing access to the plantar surface of the rat’s hind paw. Animals were allowed to acclimate in cages for 20 min before the test. A polypropylene tip (10 μL, #T-300, Axygen, Corning, NY, United States) adapted to a hand-held force transducer was positioned perpendicularly to the plantar surface of the rat’s hind paw aiming the L5 peripheral field. Then, a gradually increasing pressure (80 g maximum) was applied until animal voluntarily withdraw its paw. This mechanical stimulus was repeated up to six times separated by a 1-min interval to prevent mechanical sensitization. The withdrawal threshold was defined as the average force (g) required to animals withdraw the stimulated paw in three consistently measurements (differences < 10%). Animals were tested before and after treatments and the results (intensity of hyperalgesia) are expressed as the variation of the mechanical threshold by subtracting the baseline values (obtained before treatment) from those obtained after the treatments (Δ mechanical threshold in grams).

### Primary DRG Cultures

Dissociated DRG cell cultures were prepared as previously described (Linhart et al., 2003). Male Wistar rats (100 g) were euthanized under anesthesia and DRGs from lumbar and thoracic spine were harvested and transferred to Hank’s buffered saline solution (HBSS, #H2387, Sigma Aldrich, St. Louis, MO, United States) containing 10 mM Hepes. Cells were dissociated by incubating DRGs at 37°C in 0.28 U/mL collagenase (type II; #C6885, Sigma Aldrich, St. Louis, MO, United States) for 75 min and then in 0.25% trypsin (#T4549, Sigma Aldrich, St. Louis, MO, United States) for 12 min. After three washes with DMEM (#D5523, Sigma Aldrich, St. Louis, MO, United States) containing 10% fetal bovine serum (FBS, #10100147, Thermo Fisher Scientific, Waltham, MA, United States) and penicillin 50 U/mL + streptomycin 50 mg/mL (#15140122, Thermo Fisher Scientific, Waltham, MA, United States), cells were dissociated using a fire-polished glass Pasteur pipette. Dissociated cells were then plated in dishes coated with Matrigel (#354234, Corning, Corning, NY, United States) and the cultures were maintained in a humid 5% CO_2_ atmosphere at 37°C. Calcium experiments were performed after 3 days *in vitro* to allow the growth of satellite glial cells. The cultures media (DMEM + 10% FBS + 50 U/mL penicillin and 50 mg/mL streptomycin) was changed every other day.

### Cell Culture Treatments for Calcium Imaging

Cultures were stimulated with 2′(3′)-*O*-(4-Benzoylbenzoyl)adenosine 5′-triphosphate triethylammonium salt (BzATP, #B6396, Sigma Aldrich, St. Louis, MO, United States), the main agonist for P2X7R (Donnelly-Roberts et al., 2009). BzATP was prepared 10-fold the final concentration (1 mM solution for 100 μM final concentration) in HBSS and administered directly in the buffered solution wherein the cultures were maintained during image acquisition. Some cultures were previously incubated with A-740003 (#3701, Tocris Bioscience, Bristol, United Kingdom), a selective antagonist of P2X7R (Honore et al., 2006) for 10 min. A-740003 was diluted at final concentrations (1 μM) in HBSS and maintained in cultures during agonist stimulus. To confirm cellular viability in those cultures with non-responsive cells after the incubation with the P2X7R antagonist, a stimulus of capsaicin (10 μM final concentration) was administered at the end of the experiment.

### Intracellular Calcium Imaging

Calcium recordings were performed after 18–24 h of culture. Cells were loaded with the Ca^2 +^ indicator Fluo-3 AM 10 μM (#F23915, Thermo Fisher Scientific, Waltham, MA, United States) in HBSS containing 10 mM Hepes for 1 h. After loading, cells were washed three times and kept with HBSS as described. All drugs were administered directly in buffered solution during image acquisition and the Ca^2 +^ dynamics was recorded for 3 min after agonist addition. Fluorescence was excited at 506 nm and images of emitted fluorescence (at 526 nm) were acquired for each second by confocal microscope (Zeiss LSM510 Meta) in the Advanced Microscopy Center of ICBIM/UFU. Data are presented as ΔF/F0, where F0 is the baseline, to normalize for differences in cell loading. As the field of view contained 20–40 SGCs in the focal plane, recordings were made from several cells simultaneously.

### SGC-Enriched Cultures

Satellite glial cells were isolated from DRG cell cultures (see section Primary DRG Cultures) using a protocol adapted from England et al. (2001), Castillo et al. (2013). DRG cell cultures were kept for 4 days *in vitro* when glial cells are numerous. To isolate SGCs, 1 mL of fresh culture media (DMEM + 10% FBS + 50 U/mL penicillin and 50 mg/mL streptomycin) was added and all cells were gently detached from the culture dishes using a fire-polished Pasteur pipette. The 1 mL of cell suspension was collected and centrifuged at 3000 *g* during 5 min at room temperature to remove debris. Then 250 μL of the cell suspension was plated again on new uncoated 24-well plates (1 DRG culture was split into 4 SGC-enriched cultures). The absence of coating prevents the attachment of neurons and does not affect attachment or growth of the glial cells (Capuano et al., 2009). Also, the culture media was changed 2 h after the cells plating to remove any cell (mostly neurons) in suspension. The SGC-enriched cultures were then maintained in a humid 5% CO_2_ atmosphere at 37°C for 5–7 days *in vitro* or until the SGCs have proliferated at a confluence up to 70%. Media was changed every other day.

### Cell Culture Treatments for Cytokine Measurement

For the IL-1β synthesis and release experiments, SGCs were cultured in 24-well plates and an adapted protocol from Colomar et al. (2003), Rampe et al. (2004) was used to induce IL-1β synthesis in SGC-enriched cultures. Cultures were maintained with fresh medium (DMEM supplemented as previously described) or stimulated with lipopolysaccharides (LPS, 1 μg/mL; #L8274, Sigma Aldrich, St. Louis, MO, United States) for 24 h kept in a humid 5% CO_2_ atmosphere at 37°C. Some cultures were then incubated for 15 min with A74000 (1 μM) that was prepared 10-fold the final concentration and administered directly in cultures medium. After incubation, cultures were treated for 30 min with BzATP (100 μM) also prepared 10-fold the final concentration. All drugs were diluted in the culture medium. After treatments, the supernatant was collected and mixed with protease inhibitors (10 mM EDTA and 20 kl/ml aprotinine; Sigma Aldrich, St. Louis, MO, United States). Forthwith the cells were lysed with an ice-cold solution of Phosphate Buffer Saline (PBS) containing 0.1% Triton X-100 and protease inhibitors to obtain the cell lysates.

### Enzyme-Linked Immunosorbent Assay (ELISA)

An adaptation of ELISA (Teixeira et al., 2010) was used to determine whether P2X7R was able to regulate the synthesis and releasing of IL-1β by SGCs. The cells lysates or the supernatant were centrifuged at 10,000 rpm for 15 min at 4°C, and the supernatants were stored at −80°C for posterior use to evaluate the protein levels of IL-1β. IL-1β was quantified by Rat IL-1β/IL-1F2 DuoSet ELISA Kit (#DY501, R&D Systems, Minneapolis, MN, United States) and all procedures followed the instructions of the manufacturer. All samples were running in duplicates and procedures were repeated at least twice.

### DRG Immunohistochemistry

Animals were terminally anesthetized with ketamine (i.p. 85 mg/kg)/xylazine (i.p. 10 mg/kg) and perfused through the ascending aorta with saline solution followed by 4% paraformaldehyde (PFA, pH 7.4, 4°C). After perfusion, L5 DRG were removed and post-fixed in 4% PFA for 2 h, which was then replaced with 30% sucrose for 48 h at 4°C. Each DRG were embedded in optimum cutting temperature and sections of 14 μm were made in a cryostat using gelatinized slides. The sections were processed for immunofluorescence, first incubated in 0.1 M glycine for 30 min at room temperature, to inactivate free aldehydes, and then blocked with 2% BSA in 0.2% Triton X-100 for 1 h at room temperature. The mixture of rabbit polyclonal anti-P2X7R (1:200, #APR-004, Alomone Labs, Jerusalem, ISR), goat polyclonal anti-TRPV1 (1:100, #AF3066, R&D Systems, Minneapolis, MN, United States) and mouse monoclonal anti-glutamine synthetase (1:200, #MAB302, EMD Millipore, Darmstadt, DEU) were incubated for 2 h at room temperature. A mixture of mouse monoclonal anti-GFAP (1:100, #G3893, Sigma Aldrich, St. Louis, MO, United States) and goat polyclonal anti-IL-1β (1:100, #AF-501-NA, R&D Systems, Minneapolis, MN, United States) was incubated overnight at 4°C. Finally, a mixture of Alexa 488 (A21206), Alexa 546 (A11058) and Alexa 594 (A11058) conjugated secondary antibodies (1:1000 each, Thermo Fisher Scientific, Waltham, MA, United States) were incubated for 1 h at room temperature and then nuclei were stained with DAPI 0.25 μg/mL (#D9542, Sigma Aldrich, St. Louis, MO, United States) for 10 min. Sections were examined in the National Institute of Science and Technology in Photonics Applied to Cell Biology (INFABIC/UNICAMP) using a confocal laser-scanning microscope (Zeiss LSM780-NLO).

### RNA Extraction, cDNA Synthesis, and qPCR Experiment

RNA was extracted from whole DRG tissue by TRIZOL^®^ Reagent (#15596026, Thermo Fisher Scientific, Waltham, MA, United States) according to manufacturer instructions. Subsequently, total RNA was quantified by ultra-low volume spectrophotometer (Epoch Microplate Spectrometer, BioTek Instruments, Winooski, VT, United States) and 500 ng of RNA were used to perform cDNA synthesis (SuperScript^®^ VILO cDNA Synthesis Kit, #11755, Thermo Fisher Scientific, Waltham, MA, United States). Real-Time PCR (RT-qPCR) experiment was performed for relative quantification of P2X7R and Il-1β to an index of endogenous control genes (Arfgef1, G3bp2, Mtm8, Ppp1cc, Serpinb6) by 2^−ΔΔ*C*_*T*_^ method (see Table 1 for the primer sequences). The RT-qPCR reactions were conducted using SYBR Green as a fluorescent signal (SYBR Power Up Master Mix, #4367659, Thermo Fisher Scientific, Waltham, MA, United States). Primers were designed using Primer3 software available at NCBI’s Pick Primer tool^1^ between exons when possible and its specificity to the gene of interest was validated by comparison with the rat genome using BLAST tool from NCBI webpage^1^. Technical triplicates for all samples as well as negative controls were used for each gene.

**TABLE 1:** Primer Sequences used for RT-qPCR.

**Gene**	**Forward sequence 5′- 3′**	**Reverse sequence 5′- 3′**
P2rx7	AGTCCCTGTTCCCTGGCTACAACT	GGATGTCAAAACGCACGCCG AAGG
I l1b	TGCTAGTGTGTGATGTTCCC	AGCACGAGGCATTTTTGTTG
Arfgef1	CAACAGGTTTAAAGCTCACGCA	TCCTGTTCAGGTGGTTGTGA
G3bp2	ACCACAAGCGGAGGAGAAAC	CATCAACCCTTGGCTTTGGC
Mtmr8	GTGCATTGACAGGGAAGGTG	GCCACTCTCGCTCTATCAGG
Ppp1cc	GAGACCCTCATGTGCTCCTTC	AGGCCTGATCCAACCCGTG
Serpinb6	GAGTCTAGGGTACGTTCTGCTG	TCCATGATGGTGAACCTGCCC

### Statistical Analysis

The statistical analysis was performed using Prism v.6 (GraphPad Software Inc., La Jolla, CA, United States). Statistical significance between groups (defined as *P* < 0.05) was calculated by one-way ANOVA and Bonferroni *post hoc* test, otherwise by using the two-tailed Student’s *t*-test between two groups. Sample sizes are indicated in the figure legends and in the text. Data in the figures are expressed as mean ± S.E.M. The long line above two or more data sets indicates no statistical difference between those groups. The symbol (#) indicates statistical difference in comparison to saline-treated group (hind paw treatments). The symbol (^∗^) indicates statistical difference in comparison to vehicle-treated group (DRG treatments; ^∗^*P* < 0.05; ^∗∗^*P* < 0.01; ^∗∗∗^*P* < 0.001).

## Results

### The Selective Blockade of P2X7R in DRG Reduces the Mechanical Hyperalgesia Induced by CFA, Carrageenan, and IL-1β, but Not PGE_2_

**FIGURE 1:**
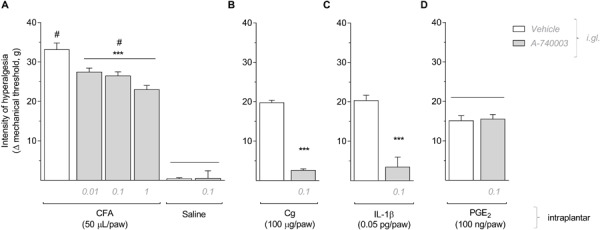
Selective blockade of P2X7R expressed on satellite glial cells reduced the mechanical hyperalgesia induced by CFA, Carrageenan or IL-1b, but not PGE2. **(A)** A-740003 (0.01, 0.1, and 1.0 mM/5 μL) administered in DRG (i.gl.) immediately before intraplantar administration of CFA significantly reduced the mechanical hyperalgesia evaluated 6 h later (One-way ANOVA and Bonferroni post-test; *P* < 0.001, *n* = 6 animals). The intraganglionar administration of A-740003 (0.1 mM/5 μL) in saline-treated animals did not change the mechanical baseline threshold. **(B)** Mechanical hyperalgesia evaluated 3 h after intraplantar administration of Carrageenan (Cg) or IL-1b **(C)** was prevented by the treatment with A-740003 (0.1 mM/5 μL) in DRG (Student’s *t*-test; *P* < 0.001, *n* = 6 animals), but it did not affect PGE2-induced mechanical hyperalgesia **(D)**. Data are expressed as mean ± SEM. A line above two data sets indicates no statistical difference between those groups; (#) indicates statistical difference comparing to the saline group; (***) indicates statistical difference comparing to the vehicle solution group.

To evaluate the participation of P2X7R expressed in DRG during the development of peripheral inflammatory hyperalgesia, different doses of the selective P2X7R antagonist A-740003 (0.01 mM, 0.1 mM or 1.0 mM/5 μL) were administered in the right L5 DRG immediately before intraplantar administration of CFA (50 μL/paw) in the ipsilateral hind paw. The electronic von Frey test was conducted 6 h later to measure the mechanical hyperalgesia. As shown in Figure 1A, A-740003 decreased the CFA-induced mechanical hyperalgesia compared to vehicle-treated group (One-way ANOVA and Bonferroni post-test; *F* = 11.84, *P* < 0.001, *n* = 6). Also, the intraganglionar administration of A-740003 (0.1 mM/5 μL) in animals treated with saline intraplantar did not change the mechanical nociceptive threshold, which excludes any alterations in the mechanical baseline. Thus, this dose was used in the following experiments. To confirm the selective effect of A-740003 (0.1 mM) on other models of inflammatory hyperalgesia, we administrated carrageenan (100 μg/paw), IL-1β (0.5 pg/paw) or PGE_2_ (100 ng/paw) in the right hind paw and measured the mechanical hyperalgesia 3 h later. A-740003 (0.1 mM/5 μL) administered in the ipsilateral L5 DRG immediately before the inflammatory stimuli prevented the hyperalgesia induced by carrageenan and IL-1β (Figures 1B,C; Unpaired *t*-test; *t* = 23.35 and *t* = 5.89 respectively, *P* < 0.001, *n* = 6), but did not affect the hyperalgesia induced by PGE_2_ (Figure 1D; Unpaired Student’s *t*-test; *t* = 0.23, *P* = 0.816, *n* = 6).

### Knockdown of the P2X7R in DRG Reduces the Mechanical Hyperalgesia Induced by CFA

**FIGURE 2:**
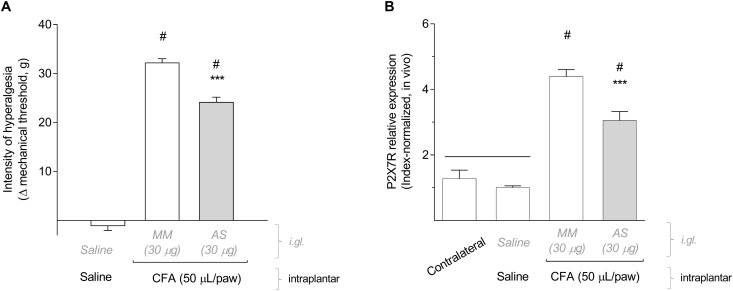
The knockdown of P2X7R in DRG reduced the mechanical hyperalgesia induced by CFA. **(A)** Antisense ODN (AS) for P2X7R (30 mg/5 ml daily for 4 days) administered in DRG before intraplantar administration of CFA significantly reduced the mechanical hyperalgesia assessed 6 h later (One-way ANOVA and Bonferroni post-test; *P* < 0.001, *n* = 6 animals). **(B)** Treatment with antisense ODN (AS) significantly reduced the CFA-induced upregulation of P2X7R expression in the injected DRG. Relative expression of P2X7R in the saline-treated DRGs did not differ from the contralateral non-treated DRGs (One-way ANOVA and Bonferroni post-test; *P* < 0.001, *n* = 5 animals). Data are expressed as mean ± SEM. A line above two data sets indicates no statistical difference between those groups; (#) indicates statistical difference comparing to the control groups; (***) indicates statistical difference comparing to the mismatch group (MM).

To knock down the expression of P2X7R in the DRG, we administered an antisense ODN for P2X7R or a mismatch sequence (MM), as control, in the right L5 DRG (30 μg/5 μl) once a day for 4 days before CFA injection (50 μL/paw) in the ipsilateral hind paw. Similarly to the selective blockade with A-740003, the P2X7R knockdown using the antisense (AS) also significantly reduced the mechanical hyperalgesia, assessed 6 h after CFA injection, when compared to the MM treatment (Figure 2A; One-way ANOVA and Bonferroni post-test; *F* = 331.9, *P* < 0.001, *n* = 6). To confirm the P2X7R knockdown, we analyzed the levels of P2X7R expression by RT-qPCR in all treated DRG (AS, MM, or saline) and in non-treated DRG (contralateral) as a control for basal expression (Figure 2B). For this 2^−ΔΔ*C*_*T*_^ analysis, we used an index of endogenous controls (Serpinb6, Mtmr9, Arfgef1, and G3bp2). The relative expression of P2X7R in the AS-treated DRG was significantly reduced compared to the MM treatment. However, in the MM-treated DRG the expression of P2X7R was already 4-fold increased after 6 h of the CFA inflammation (One-way ANOVA and Bonferroni post-test; *F* = 58.14, *P* < 0.001, *n* = 5). The expression of P2X7R in the saline-treated DRG did not differ from the basal expression in the contralateral DRG. This result demonstrates the P2X7R expression was not altered solely by the repeated intraganglionar injections.

### P2X7R Are Expressed by Satellite Glial Cells in DRG and Can Be Functionally Activated and Blocked *in vitro*

**FIGURE 3:**
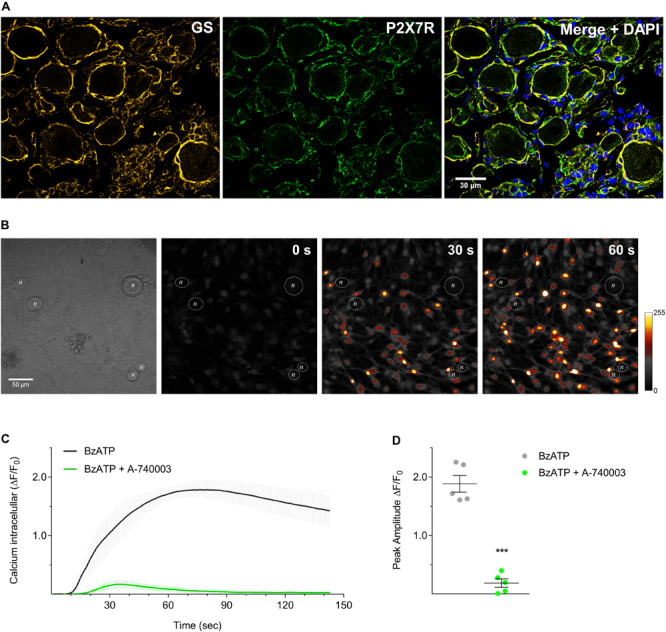
P2X7R are expressed by SGCs in DRG and can be functionally activated and blocked in culture. **(A)** Co-staining of glutamine synthetase (GS, yellow) and P2X7R (green) in DRG sections of CFA-treated animals. In non-inflammatory condition, expression of P2X7R is very low and hardly detected by DRG immunohistochemistry. The merged picture shows co-localization of P2X7R and glutamine synthetase in SGCs surrounding neurons, and cell nuclei were staining with DAPI (blue). **(B)** BzATP (100 μM) induced intracellular calcium surge in cultured SGCs (colored artificially) but not in neurons (*n*). **(C)** Pretreatment with A-740003 (1 μM, green line) blocked the BzATP-induced intracellular calcium surge (gray line). **(D)** Maximum values of ΔF/F_0_ for both response curves showed in **(C)**. Data are expressed as mean ± SEM for *n* = 5 cultures/group, 20–40 SGCs sampled per culture. Symbol *** indicates statistical difference between the response peaks (Unpaired *t*-test; *P* < 0.001).

As shown in Figure 3A, the immunohistochemistry co-staining P2X7R and glutamine synthetase (GS, a glial-specific enzyme) in paraformaldehyde-fixed rat DRG sections confirmed that P2X7R are expressed on SGCs, but not on DRG neurons, after CFA inflammation. Expression of P2X7R in non-inflammatory conditions is very low (as shown in Figure 2B) and hardly detected by immunohistochemistry of the DRG (data not shown). To check P2X7R activation and blockade in SGCs, we evaluated changes in intracellular calcium concentration by confocal microscopy using mixed DRG cell cultures from adult rats. The P2X7R was activated by its agonist BzATP (100 μM) which increased the intracellular calcium concentration in SGCs but not in neurons (Figure 3B). The incubation with P2X7R selective antagonist A-740003 (1 μM), added to cultures 5 min before BzATP, blocked the BzATP-induced intracellular calcium surge in SGCs (Figures 3C,D; Unpaired Student’s *t*-test; *t* = 10.70, *P* < 0.001, *n* = 5 cultures/group, 20–40 SGCs sampled per culture).

### Activation of P2X7R Increases the Release but Not the Synthesis of IL-1β in Satellite Glial Cells After an Inflammatory Stimulus

**FIGURE 4:**
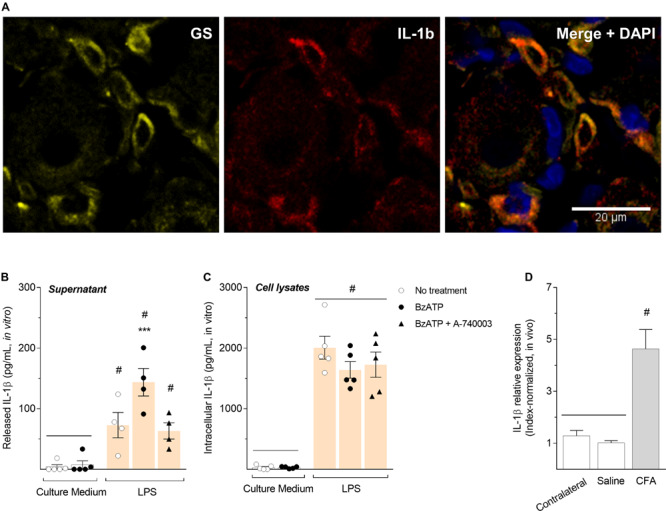
Activation of P2X7R increased the release but not the synthesis of IL-1β in satellite glial cells after an inflammatory stimulus. **(A)** Co-staining of glutamine synthetase (GS, yellow) and IL-1β (red) in DRG sections. In non-inflammatory condition, expression of IL-1β is very low and hardly detected by DRG immunohistochemistry. The merged picture shows co-localization of IL-1β and glutamine synthetase in SGCs surrounding neurons, and cell nuclei were staining with DAPI (blue). **(B)** SGC-enriched cultures were stimulated with LPS (1 μg/mL) for 24 h and then incubated with A-740003 (1 μM) for 15 min followed by treatment with BzATP (100 μM) for 30 min. LPS induced the release of IL-1β in the supernatant of SGCs cultures, but BzATP significantly increased the release of IL-1β by SGCs, which was prevented with A-740003. **(C)** IL-1β synthesis is increased *in vitro* only in LPS-stimulated cultures, regardless of P2X7R activation or blockade, and **(D)** *in vivo* after 6 h of the CFA inflammation in the ipsilateral hind paw. IL-1β relative expression in the contralateral paw had no difference from the saline group. Data are expressed as mean ± SEM. Symbol # indicates statistical difference comparing to control groups and *** indicates statistical difference comparing to LPS-stimulated groups [One-way ANOVA and Bonferroni post-test; *P* < 0.001, *n* = 4-5 cultures in **(B,C),** *n* = 5 animals in **(D)**].

To confirm SGCs express IL-1β, we performed an immunohistochemistry co-staining IL-1β (both precursor and mature forms) and glutamine synthetase (GS) in paraformaldehyde-fixed rat DRG sections. Our results showed that IL-1β is expressed on SGCs after CFA-induced inflammation (Figure 4A), in addition to P2X7R (Figure 3A). To evaluate whether the release of IL-1β in DRG is regulated by activation of P2X7R during the peripheral inflammation, we used an *in vitro* approach. SGC-enriched cultures isolated from DRG were maintained with fresh culture medium (controls) or stimulated with LPS (1 μg/mL) for 24 h. Cultures were then incubated with A-740003 (1 μM) for 15 min followed by treatment with BzATP (100 μM) for 30 min. Supernatant and cell lysates were collected and the levels of IL-1β were measured by ELISA. Our data show P2X7R activation by BzATP without an inflammatory stimulus did not induce IL-1β release by SGCs (Figure 4B, white bars). However, after the LPS inflammatory stimulation, the release of IL-1β was induced into the supernatant of SGCs cultures. Then, P2X7R activation by BzATP significantly increased the release of IL-1β by LPS-stimulated SGCs cultures, which was prevented by the P2X7R blockade with A-740003 (Figure 4B, colored bars; One-way ANOVA and Bonferroni post-test; *F* = 16.60; *P* < 0.001, *n* = 5).

Further, we investigated the intracellular levels of IL-1β in the SGCs cultures, to test whether activation of P2X7R is relevant also for IL-1β synthesis. Our results show that intracellular levels of IL-1β were only increased with the LPS inflammatory stimulation, regardless of P2X7R activation or blockade (Figure 4C; One-way ANOVA and Bonferroni post-test; *F* = 48.12, *P* < 0.001, *n* = 5). We also analyzed the expression of IL-1β by RT-qPCR in the L5-DRG of rats treated with CFA (50 μL/paw) or saline, along with non-treated DRG (contralateral) as a control for basal expression. The relative expression of IL-1β was also increased *in vivo* after 6 h of the CFA inflammation (Figure 4D; One-way ANOVA and Bonferroni post-test; *F* = 20.05, *P* < 0.001, *n* = 5). The expression of IL-1β in the saline-treated DRG did not differ from the basal expression in the contralateral DRG; both levels were very low and hardly detected also by DRG immunohistochemistry (data not shown).

## Discussion

Although satellite glial cells (SGCs) have no projections outside the sensory ganglia, in this study, we detected a major process occurring at the dorsal root ganglion (DRG) involving the active participation of SGCs in the development of peripheral inflammatory hyperalgesia. Peripheral inflammatory hyperalgesia is the sensitization of the peripheral terminals of nociceptors due to inflammation. It has been established that administration of inflammatory agents such as the complete Freund’s adjuvant (CFA), carrageenan, or lipopolysaccharide (LPS), leads to an upregulation of proinflammatory mediators, including interleukin-1 beta (IL-1β), in the local tissue (Cunha et al., 2000; Verri et al., 2006; Ren and Torres, 2009). IL-1β as a mediator of the inflammatory hyperalgesia induces the upregulation of cyclooxygenase-2 (COX-2) and stimulates the subsequent release of COX products, such as prostaglandin E2 (PGE_2_). PGE_2_ is the main mediator responsible for nociceptive sensitization that causes inflammatory hyperalgesia in both peripheral (Ferreira, 1981; Verri et al., 2006) and the CNS (Samad et al., 2001).

Using a technique to administer drugs directly into the DRG (right L5), we selectively inhibited P2X7R activation in SGC by injecting the selective P2X7R antagonist A-740003 (Honore et al., 2006) or the antisense oligonucleotides knocking down P2X7R expression. The selective blockade of P2X7R expressed on SGCs prevented the mechanical hyperalgesia induced by carrageenan or IL-1β. Also, P2X7R blockade or knockdown significantly reduced the mechanical hyperalgesia assessed 6 h after the CFA administration. Undoubtfully, P2X7R stands out among other purinergic receptors for playing a central role in inflammatory and neuropathic pain-like behavior (Dell’Antonio et al., 2002; Chessell et al., 2005; McGaraughty et al., 2007; Carroll et al., 2009; Honore et al., 2009; Arulkumaran et al., 2011; Luchting et al., 2016; and for review see Savio et al., 2018; Zhang et al., 2020). Besides, studies suggest that P2X7R expressed particularly in DRG have a role for the neuropathic pain-like behavior associated with HIV glycoprotein 120 (Wu et al., 2017), visceral hyperalgesia (Liu et al., 2015), and acute nociception (Lemes et al., 2018). In the present study, we describe for the first time the active participation of P2X7R expressed on SGCs on the establishment of peripheral inflammatory hyperalgesia.

However, we have shown that the selective blockade of P2X7R in DRG did not affect the PGE_2_-induced hyperalgesia. As a final inflammatory mediator, PGE_2_ acts on EP receptors and directly stimulates primary afferent neurons. Thus, the administration of PGE_2_ at a pharmacological dose of 100 ng/paw induces hyperalgesia (Prado et al., 2013). But the model of PGE_2_ inducing hyperalgesia lacks important upstream events, such as the IL-1β signaling, which is a shared event between the other models of hyperalgesia we used – CFA, carrageenan, and IL-1β (Cunha et al., 2000; Ren and Torres, 2009). In addition to inducing the synthesis of PGE_2_, IL-1β can also play as a final inflammatory mediator acting at nociceptors, a mechanism that substantially increases neuronal excitability in pain signaling (Cunha et al., 2000; Binshtok et al., 2008; Dinarello, 2018). Therefore, we suggest that a mechanism dependent on IL-1β, but not prostaglandins, could be promoting the somatic release of ATP in DRG and leading to the activation of P2X7R on SGCs.

Our results revealed the constitutive expression of P2X7R in DRG, and the immunofluorescence confirmed the expression of P2X7R in SGCs. However, P2X7R expression was considerably increased (4-fold) after the intraplantar administration of CFA. Other studies, mostly in neuropathic pain-like behavior models, also detected upregulation of P2X receptors in trigeminal SGCs (Kushnir et al., 2011) and specifically upregulation of P2X7R in spinal microglia (Kobayashi et al., 2011; He et al., 2012; Li et al., 2017), and Schwan cells (Song et al., 2015). ATP-induced purinergic signaling is used by glial cells for intercellular communication in both central (Butt, 2011; Verderio and Matteoli, 2011; Puerto et al., 2013) and peripheral nervous system (Gu et al., 2010; Hanani, 2012; Huang et al., 2013). In neurons, ATP accumulates in vesicles near the presynaptic membrane and is released by exocytosis as a neurotransmitter (Burnstock, 2006; Lazarowski, 2012). Particularly in the sensory ganglia, studies have already suggested that vesicles holding ATP can be released by the cell body of sensory neurons following activation with capsaicin (Matsuka et al., 2001) or electrical stimulation (Zhang et al., 2007). Increasing evidence show ATP as a major transmitter released in the sensory ganglia (for review see Kobayashi et al., 2013; Goto et al., 2017); and intercellular communication via purinergic signaling appears to be an essential mechanism for pain (Ding et al., 2000; Toulme et al., 2010; Kobayashi et al., 2013; Burnstock, 2016; Lemes et al., 2018; Magni et al., 2018). Therefore, the upregulation of P2X7R in SGCs could be relevant for boosting intercellular communication in DRG during inflammatory hyperalgesia.

A potential role for P2X7R from SGCs during inflammatory hyperalgesia is the regulation of IL-1β release in DRG. IL-1β is produced from an inactive precursor protein, pro-IL-1β (31 kDa), that remains accumulated at the intracellular compartment. For the IL-1β maturation, a multiprotein complex called inflammasomes is recruited by mechanisms yet not fully understood to cleave the pro-IL-1β into the active form (17 kDa) and ensure its following secretion (Brough and Rothwell, 2007; Garlanda et al., 2013; Malik and Kanneganti, 2017). Several reports emphasize that ATP-mediated P2X7R activation is essential for rapid maturation and release of IL-1β from activated immune cells, including macrophages and microglial cells (Ferrari et al., 2006; Clark et al., 2010; Stoffels et al., 2015; Giuliani et al., 2017). In the present study, we also verified a connection between P2X7R activation and IL-1β release from SGCs after inflammatory stimulation. In SGC-enriched cultures treated with LPS, the activation of P2X7R by BzATP induced the release of IL-1β in the supernatant, which was blocked by A-740003. However, the activation of P2X7R without the inflammatory stimulus did not provoke the release of IL-1β or the synthesis of the pro-IL-1β. These results might indicate the involvement of caspases proteins which participates in the IL-1β maturation (Maelfait et al., 2008).

We found that SGCs activated by the inflammatory stimuli show an increased expression of IL-1β both *in vivo* and *in vitro* through a mechanism independent of P2X7R activation. Likewise, other studies have also reported the upregulation of IL-1β in SGCs in consequence of peripheral inflammation or nerve injury (Guo et al., 2007; Takeda et al., 2007; Araldi et al., 2013). Although P2X7R activation has a critical role on IL-1β release, there is no evidence on P2X7R modulation on the expression of the Il-1β precursor (Chessell et al., 2005; Clark et al., 2010; Giuliani et al., 2017). The synthesis of pro-IL-1β is primarily induced by pathological conditions and normally involves activation of toll-like receptors (TLRs) (Dinarello, 2018). In our *in vitro* approach, LPS itself could be triggering the synthesis of the IL-1β precursor in SGCs through direct stimulation of the toll-like receptors 4 (TLR4) (Tse et al., 2014). However, further investigation is required to understand the mechanism underlying the increase of pro-IL-1β synthesis in DRG *in vivo*. Possibly, different neuronal mediators might be involved to induce IL-1β synthesis in SGC. Sensory neurons in response to electrical or chemical stimulation can release substance P or glutamate concurrent with ATP (Matsuka et al., 2001; Dissing-Olesen et al., 2014). Also, trigeminal cells in culture stimulated with IL-1β release calcitonin gene-related peptide (CGRP) through a mechanism dependent on COX-2-induced PGE_2_ synthesis (Neeb et al., 2011). Because SGCs have receptors for CGRP (De Corato et al., 2011), substance P (Matsuka et al., 2001) and NMDA (Ferrari et al., 2014), these neuronal mediators could also participate in neuron-glia signaling in the DRG.

In summary, our study has provided evidence for a specific role of P2X7R in neuronal soma-glia communication associated with the regulation of the IL-1β release in DRG during peripheral hyperalgesia. Our data suggest that inflammatory mediators released at the peripheral level lead to ATP release at the DRG, activating P2X7R at SGC and triggering the release of IL-1β. At the DRG, IL-1β was shown to upregulate cyclooxygenase activity and thus inducing prostaglandin production (Araldi et al., 2013). Prostaglandins are responsible for neuronal sensitization, contributing to the development and maintenance of the inflammatory hyperalgesia. Therefore, peripheral inflammation appears to induce a further inflammatory response at the sensory ganglia that might maintain or amplify neuronal sensitization, increasing pain-like behavior.

This study draws attention to the sensory ganglia as an important pharmacological target and can be used to support the development of new treatments for inflammatory diseases. We have described a pharmacological mechanism of analgesia that is independent of COX inhibition and targets structures outside the CNS. Also, the ganglionic injection is a technique, *per se*, translational. In humans, it has been used to treat mainly patients with chronic pain (Sapunar et al., 2012). Here we used this technique as a methodological tool to investigate hyperalgesia-related mechanisms restrict to the DRG. It’s noteworthy that the DRG is only partially covered by the dura mater, thus showing good bioavailability to drugs acting in the peripheral nervous system (Esposito et al., 2019). Therefore, DRG could be targeted by the systemic administration of drugs that do not cross the blood-brain barrier. Treatments using those classes of drugs would have reduced side effects without the limitations of using ganglionic injections.

## Data Availability Statement

All datasets generated for this study are included in the manuscript.

## Ethics Statement

The animal study was reviewed and approved by Committee on Animal Research of the University of Campinas (3022-1).

## Author Contributions

AN, CL, and CP conceived the project and designed the study. AN, FF, and SM acquired the data. AN, DA, CT, CS, and CL analyzed and interpreted the data. AN, MP, CL, and CP drafted the manuscript. AN, FF, SM, DA, MP, CT, CS, CL, and CP approved the final version to be submitted.

## Conflict of Interest

The authors declare that the research was conducted in the absence of any commercial or financial relationships that could be construed as a potential conflict of interest.

## Footnotes

1 www.ncbi.nlm.nih.gov/tools/primer-blast
